# An Overview of Hardware for Protein Crystallization in a Magnetic Field

**DOI:** 10.3390/ijms17111906

**Published:** 2016-11-16

**Authors:** Er-Kai Yan, Chen-Yan Zhang, Jin He, Da-Chuan Yin

**Affiliations:** Institute for Special Environmental Biophysics, Key Laboratory for Space Bioscience and Space Biotechnology, School of Life Sciences, Northwestern Polytechnical University, Xi’an 710072, China; yerkai@mail.nwpu.edu.cn (E.-K.Y.); zhangchenyan@nwpu.edu.cn (C.-Y.Z.); hej@mail.nwpu.edu.cn (J.H.)

**Keywords:** protein crystallization, magnetic field, apparatus, superconducting magnet

## Abstract

Protein crystallization under a magnetic field is an interesting research topic because a magnetic field may provide a special environment to acquire improved quality protein crystals. Because high-quality protein crystals are very useful in high-resolution structure determination using diffraction techniques (X-ray, neutron, and electron diffraction), research using magnetic fields in protein crystallization has attracted substantial interest; some studies have been performed in the past two decades. In this research field, the hardware is especially essential for successful studies because the environment is special and the design and utilization of the research apparatus in such an environment requires special considerations related to the magnetic field. This paper reviews the hardware for protein crystallization (including the magnet systems and the apparatus designed for use in a magnetic field) and progress in this area. Future prospects in this field will also be discussed.

## 1. Introduction

Growing protein crystals of high quality is an important issue because high-quality protein crystals are useful in a number of research fields. For instance, high-quality protein crystals are usually required for determining the molecular structure of the protein. It is especially necessary to obtain high-quality crystals when high-resolution structure information is needed. High-quality protein crystals can also be used as the starting materials for preparing cross-linked protein crystals, which might be used in applications such as biosensors or catalysts.

The quality of protein crystals is determined by nucleation and growth processes, which are affected by physical, chemical and biochemical factors. To obtain high-quality protein crystals, methods involving physical, chemical and biochemical factors may be applied. Modifying or controlling the physical environment is a simple means to affect the processes of crystallization in order to fine-tune the crystal quality. Therefore, many studies have been conducted to take advantage of aspects of the physical environment such as temperature, magnetic field, electric field, microgravity, ultrasound, and high pressure. Among these physical environments, a magnetic field might lead to protein crystals with improved quality. There have been many studies on the effects of magnetic fields on protein crystallization. The results showed that magnetic fields can affect crystal quality, and the reported data showed quality improvement in most cases.

There are a few excellent reviews [1,2,3,4,5] on this subject. Wakayama [2] reviewed the phenomena of protein crystallization under a magnetic field, including magnetic orientation, a low-gravity environment, the damping of natural convection and an increase in viscosity. Vergara et al. [3] summarized the effects of physical aspects on protein crystallization using an advanced reduced-gravity facility. Sazaki [4] summarized the effects of homogeneous and inhomogeneous magnetic fields on protein crystallization, as well as the mechanisms for crystal orientation and suppression of buoyancy convection in a magnetic field. More recently, Yin [5] produced a detailed review of progress on the influence of a magnetic field on protein crystallization, including its effects on protein solutions, protein crystallization and crystal quality. All of these studies showed that a magnetic field may provide a valuable tool to obtain high-quality protein crystals. Thus, exploration of the applications of magnetic fields promises to be rewarding.

Studies using magnetic fields in protein crystallization strongly rely on hardware, e.g., the magnetic field system and the apparatus in the magnetic field that is used to provide a stable and controllable environment, to analyze the effect of the magnetic field on protein crystallization. Hence, the design and preparation of the research hardware are very important. In this review, we provide an overview of the hardware used to study the influence of a magnetic field and evaluate its benefits, if any. We also provide an overview of how the hardware might evolve so that it may be of use in the field of biocrystallogenesis in the future.

## 2. Magnet Systems That Provide a Strong and Stable Magnetic Field

The magnets used for protein crystallization can be categorized into four types: electromagnets, permanent magnets, superconducting magnets, and hybrid magnets. Table 1 lists the types of magnets and their flux densities.

### 2.1. Electromagnets

The electromagnet, a device that generates an electromagnetic signal in an energized state, provides a steady and homogeneous magnetic field for protein crystallization. The strength of the magnetic field generated by an electromagnet is lower than that of a superconducting magnet. However, the direction and magnitude of the magnetic force can be adjusted by changing the intensity and direction of the current. The magnetic field immediately disappears once the electricity is turned off. The magnetic field generated by an electromagnet has been used in protein crystallization for several decades. The first electromagnet (NPS 60 TS, Nihon Kohmitsu Co., Ltd., Nagoya, Japan) for protein crystallization has a static magnetic field of 0.1–1.6 T (homogeneity ±1%). The gap between the two poles for a deposited sample is 12 mm. Using this electromagnet, Wakayama conducted a quantitative study on lysozyme crystallization kinetics under the same electromagnetic field [12]. The study showed that the sedimentation rate of the crystals was a function of crystal size; crystals sedimented to the vessel bottom when their size reached 2–6 µm.

Later, a similar experimental setup was designed by Sakurazawa et al. [13], wherein a steady magnetic field (up to 1.1 T, homogeneity ±1%) produced by the electromagnet was used to study the orientation of ribonuclease A, lysozyme and met-myoglobin crystals using a batch method. In addition, the authors discussed the protein molecules and unit cell possessing of the magnetic anisotropy as well as the requirement for protein molecule assembly into a regular array to form the crystal orientation. Tanimoto and his co-workers [14] conducted research into the orientation of lysozyme crystals in a horizontal magnetic field provided by an electromagnet (Tokin, SEE10) and a superconducting magnet (Oxford, SM-1000). They showed that the *c*-axis of 90% of crystals was parallel to the magnetic field direction at 0.64 T. Lubbert et al. [6] investigated the mosaicity of lysozyme crystals growing inside and outside of a magnetic field (0–2.4 T) with or without gel. The magnetic field was produced by a Brucker CE-45 electromagnet, and the crystallization vessels were located in the electromagnet gap. A novel diffraction technique was invented to detect the spatial array of mosaic blocks of crystal using two point detectors and a MAR CCD area detector. The results showed that the mosaicities were anisotropic and that the influence of a magnetic field on the mosaicity of lysozyme crystals was very small.

**Figure 1:**
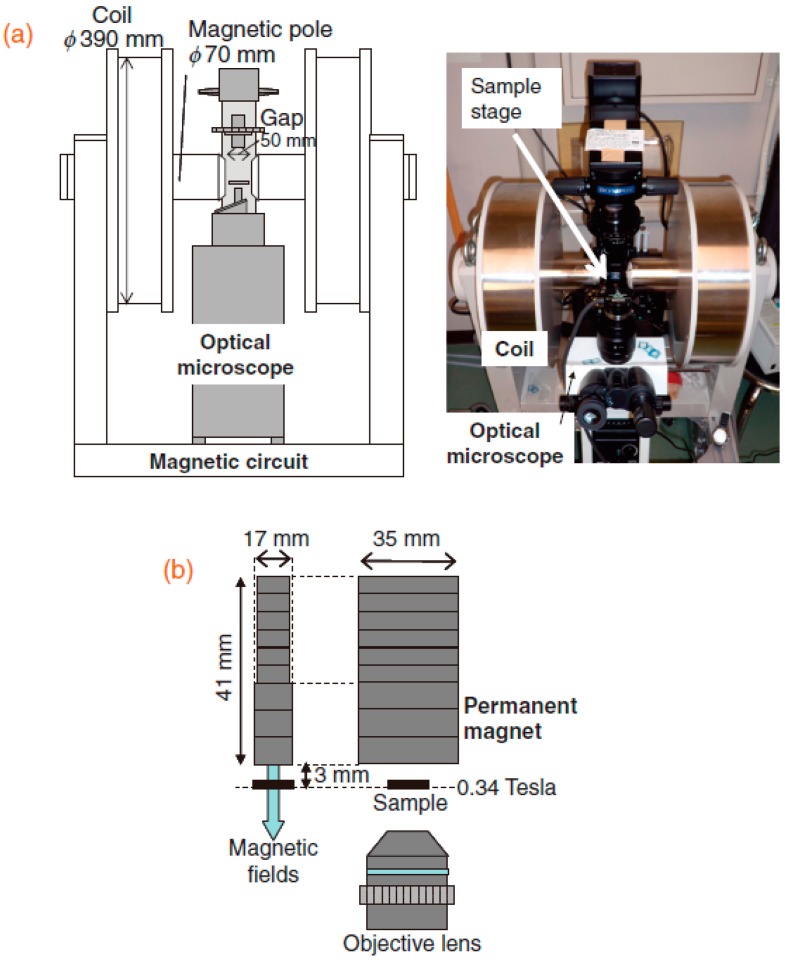
Experimental setup utilized to observe the orientation of crystal boards: (**a**) horizontal magnetic field generated by an electromagnet; and (**b**) vertical magnetic field generated by a permanent magnet [15]. Reproduced from reference [15] with permission from the Japan Society of Applied Physics.

In recent years, Iwasaka et al. [15] constructed vertical magnetic fields of 340 mT generated by a resistive electromagnet and horizontal magnetic fields of 400 mT generated by a permanent magnet, as shown in Figure 1. They showed that the horizontal magnetic fields directed the directional length of the crystals’ broadest surface, while the vertical magnetic fields directed the directional width of the crystals’ broadest surface. The combination of a horizontal and a vertical magnetic field led to a rapid orientation of crystals.

### 2.2. Permanent Magnets

A permanent magnet, as a hard magnetic body, is not easy to magnetize or demagnetize, and thus can maintain long-term magnetic stability. Permanent magnets can be classified into groups including NdFeB permanent magnets, ferrite permanent magnets, and rubber permanent magnets. The merits of permanent magnets are their low cost and stability for long-term experiments. However, the magnetic field strength is usually weak, and it is difficult to adjust the magnitude and direction of the magnetic field.

A permanent magnet (NEOMAX, 8 × 10.7 × 10 cm^3^), invented by Sumitomo Special Metal Co., Ltd., (Osaka, Japan) possesses a steep horizontal magnetic field gradient (64 T^2^/m) and high magnetic intensity (1.2 T, inhomogeneity of ±0.5%). Using this magnet, a comparison of crystal growth was conducted under a homogeneous magnetic field generated by an electromagnet (0.6 and 1.2 T) with a gradient magnetic field generated by a superconducting magnet (10 T) [16]. The results showed that the crystal number decreased when 5% of the force was added in opposition to normal gravity, while the crystal number increased when 5% of the force was added in line with normal gravity. Other permanent magnets (1.25 T, 25 × 9 × 9 mm^3^), which were composed of an Nd-Fe-B alloy (Goudsmit Company, Neuville-en-Ferrain, France), were used by Astier et al. to study crystal orientation in a magnetic field [7]. The authors showed that lysozyme and bovine pancreatic trypsin inhibitor crystals were oriented, while porcine pancreatic α-amylase crystals that underwent heterogeneous nucleation stuck at the bottom of the crystallization cell and thus were not oriented. These experiments were simple, easily performed in a laboratory and required no special apparatus.

### 2.3. Resistive Magnets

A representative resistive magnet is the water-cooled resistive magnet [8]. The maximum current of this magnet is up to 40 kA at a working voltage of 500 V. This magnet has a bore 32 mm in diameter, and its magnetic field intensity is as great as 33 T for an extended period. A strong advantage of this magnet is its low cost (water-cooled) and extremely high magnetic field intensity. Heijna et al. [17] studied the growth of diamagnetic lysozyme crystals by tuning the gravity strength from 0.15 to 1 g using this magnet. They reported that convection could be damped, stopped and reversed during this process. Furthermore, a hybrid magnet system with a 45 T magnetic field strength (with a 32 mm bore) was constructed by the High Field Magnet Laboratory [9].

### 2.4. Superconducting Magnets

#### 2.4.1. Common Superconducting Magnets

The superconducting magnet represents a breakthrough in magnetic field research and plays a role in protein crystallization. The magnetic field intensity of a superconducting magnet is very stable and can provide a stable environment for the growth of protein crystals. A remarkable advantage of the superconducting magnet is its large magnetic field intensity and gradient. Natural convection is decreased by the Lorentz force and magnetic force; thus, the magnetic field facilitates the three-dimensional regular arrangement of protein molecules. Representative superconducting magnets are shown in Table 2. Simulations of natural convection on protein crystallization in a magnetic field have also verified this phenomenon [18,19,20,21]. However, the maintenance charge of a superconducting magnet is always very expensive and usually requires a refrigerant medium (such as liquid nitrogen or liquid helium) to maintain the high magnetic intensity, which sharply increases the cost.

Superconducting magnets have been used in protein crystallization. In 1996, Watanabe et al. [22] designed an 11 T liquid-helium-free superconducting magnet in vacuum that used high-temperature superconducting current leads. The magnet used multifilamentary NbTi superconducting wire as an outer solenoid coil and Nb_3_Sn superconducting wire as an inner epoxy-impregnated coil. The cooling system consisted of two 4 K GM cryocoolers, and the magnetic field could function continuously at 10.5 T for 24 h at 4.8 K. Sazaki et al. [23] demonstrated that the magnetic field produced using this superconducting magnet system reduced crystal nucleation, oriented crystals and modified crystal behavior. This system provided an effective method to grow large crystals in small quantities. Using the same magnet, Yanagiya et al. further investigated the mechanism of lysozyme crystal orientation in a magnetic field [24]. They found that the orientation degree of lysozyme crystals greatly depended on the rate of crystal growth, the geometry of the container and the intensity of the magnetic field.

A static magnetic field produced by a cryogen-free superconducting magnet (8 T, Sumitomo Heavy Industry, Tokyo, Japan) was used to grow an oriented microcrystal array [25]. In this experiment, the lysozyme microcrystals were oriented in a polymer matrix under a magnetic field, and the alignment was consolidated by UV light irradiation. Then, these microcrystals were diffracted using traditional X-ray diffraction technology with a diffraction resolution up to 3.0 Å. This design provided a novel method to analyze the three-dimensional arrangement of proteins, and the crystal size required for this technology was not as large as that required for the conventional single-crystal X-ray diffraction method.

**Figure 2:**
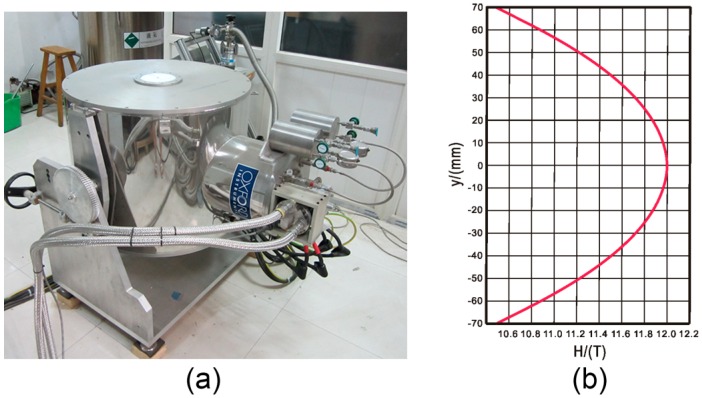
Setup used for high-throughput crystallization: (**a**) the front view of the transverse magnet; and (**b**) the magnetic flux density over ±70 mm at full field.

Another typical example is the cryogen-free superconducting magnet (OXFORD Instruments, Abingdon, UK) designed by Oxford instruments, as shown in Figure 2. Its maximum central magnetic field is 12 T, and it has a bore 15 mm in diameter. This magnet is stable both thermally and physically under high Lorentz forces, and it can operate in a “persistent mode”. Huang et al. [26] invented a high-throughput protein crystallization screening instrument for use in cryogen-free superconducting. The crystals grew in capillary tubes deposited in an identical magnetic field, and they could be directly diffracted by X-ray diffraction. Diffraction data at 2.8 Å for lysozyme crystals were obtained using in situ diffraction in a home facility.

Japan Magnet Technology, Inc. was established in 1989 to focus on the development of a superconducting magnet. Subsequently, many excellent magnets were produced. A representative example is a superconducting magnet (JMTD-5 T 300 M) with a bore of *Φ*300 mm × 780 mm, whose intensity and magnetic field direction could change continuously from 0 T to 5 T and from 0° to 90°, respectively. Based on the magnetic field, Zhao et al. [10] found that the growth rate of l-alanine crystals ({120} and {011} faces) decreased or increased when experiencing an upward or downward magnetic force using this superconducting magnet, which may have been due to variation in the solution convection rate.

Another homogeneous and static magnetic field was generated by a liquid-helium-free superconducting magnet (JMTD-10T100M, Japan Magnet Technology, Inc.), which generated a high-intensity magnetic field of 10 T specifically designed for protein crystallization. Sato et al. [27,28] investigated the effects of a magnetic field on crystal quality using the device at 10 T and showed that crystal resolution was significantly improved. The same equipment was used by Lin et al. [29] and Kinoshita et al. [31] to obtain high-resolution human estrogenic 17 β-hydroxysteroid dehydrogenase and snake muscle fructose-1,6-bisphosphatase crystals and bovine adenosine deaminase crystals, respectively. In addition, the influences of magnetic fields on paramagnetic or diamagnetic materials have been of interest to researchers. In 2003, Yin et al. [32,33] analyzed the symmetry, morphology, and orientation of lysozyme crystals using three paramagnetic salts (NiCl_2_, CoCl_2_ and MnCl_2_) as precipitants. They obtained large, perfect lysozyme crystals when using NiCl_2_ and MnCl_2_ as precipitants and found that the growth rate of {011} faces was faster than that of {101} faces when using NiCl_2_ as a precipitant. In addition to the magnetic field alone, Sazaki et al. [34] used the same experimental apparatus to analyze the combined effect of a magnetic field and electric field on lysozyme crystallization. They showed that the crystal size and ratio of the orientated crystals were markedly improved when magnetic and electric fields were simultaneously applied. Saijo et al. [35] analyzed the three-dimensional structure of lysozyme grown in this high magnetic field. They showed that the crystal structure remained similar, except for a few residues, and that a higher resolution crystal could be obtained in a magnetic field.

In 2002, Japan Magnet Technology, Inc. merged with Kobe Steel’s superconducting wire business to form a new company named Japan Superconductor Technology, Inc. (JASTEC, Kobe, Hyogo, Japan). JASTEC is dedicated to the production of high-quality products related to superconducting wire and superconducting magnets, such as high-field research magnets, NMR magnets, and cryogen-free magnets. Two representative magnets produced by this company are the LH15T40 and the LH16T50.

The LH15T40 magnet consists of Nb_3_Sn and NbTi superconductors, and all the superconducting coils are connected in series. The magnet, with a laboratorial size could generate a magnetic field gradient of up to 150 T/m and can run in persistent mode without supplying current [41]. Some protein crystallization studies used this superconducting magnet device due to its wide distribution of a magnetic field. Yin et al. [42] obtained lysozyme crystals at quasi-microgravity for the first time in 2004 and showed that a quasi-microgravity environment could effectively improve the crystal quality. Subsequently, Wakayama et al. [43] investigated orthorhombic lysozyme crystallization in pseudo-microgravity utilizing the same magnet to simulate the microgravity environment in space. Nakamura et al. [40] recently found that three of fifteen protein crystals showed a magnetic orientation and that the quality of five proteins was increased compared with those obtained in a magnetic field gradient using the same magnetic field.

**Figure 3:**
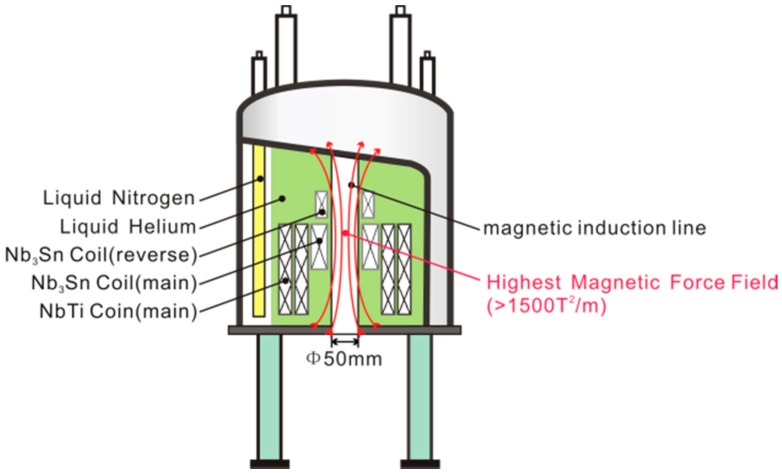
Schematic diagram of the superconducting magnet (JMTA-16T, JASTEC, Inc.) now installed at Northwestern Polytechnical University, Xi’an, China [11].

**Figure 4:**
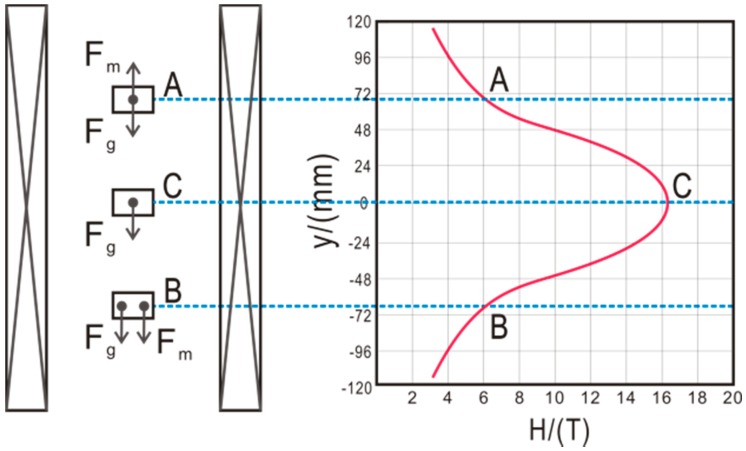
Distribution diagram of the gravitational force and Lorentz force of an object inside the superconducting magnet (JMTA-16T). Positions A, C, and B correspond to µg, 1 g and 2 g, respectively [11].

The LH16T50 superconducting magnet also consists of one Nb_3_Sn coil and two NbTi coils. The maximum central magnetic field is as great as 16.12 T, and the strongest magnetic force field is as great as 1500 T^2^/m [38], as shown in Figure 3. A disadvantage of this magnet is its requirement for continuous liquid nitrogen and helium to maintain low-temperature superconduction. Figure 4 shows the distribution of the gravitational force and Lorentz force of an object inside this superconducting magnet. Using such a magnet, Yin et al. [11] showed that high-quality lysozyme crystals could be grown in container-less conditions [44]. They found that the orientation of multiple crystals occurred when they used different paramagnetic salts (CoCl_2_ and NiCl_2_) as precipitants at different concentrations [39]. In 2013, Numoto et al. [37] reported the orientation of membrane protein (light-harvesting complex 2) crystals under high magnetic force fields for the first time; the R value and mosaicity of the crystals grown with this magnet were significantly improved compared with controls. In 2013, Cao and her co-workers [36] compared the quality of protein crystals grown in three container-less environments (agarose gel, silicone oil and diamagnetic levitation) and concluded that the diamagnetic levitation technique had the greatest benefits for obtaining high-quality protein crystals.

#### 2.4.2. Nuclear Magnetic Resonance (NMR) Magnets

Nuclear magnetic resonance (NMR) instruments, commonly used for disease surveillance, have recently attracted substantial attention as the source of a static magnetic field for protein crystallization. Moreno et al. [45] grew highly perfect single-protein crystals (thaumatin, lysozyme and ferritin) in the presence of gels and magnetic fields (Oxford and Varian magnets with current frequencies of 500 and 300 MHz, respectively). During the experiments, capillaries were filled with protein solutions, and all the capillaries were placed in an NMR tube with silica hydrogel. In addition, Moreno et al. [46] obtained high-quality lysozyme crystals, in terms of mosaicity and diffraction resolution, under the same magnetic fields. They showed that the crystal quality was remarkably improved due to the suppression of convective transport and the control of nucleation kinetics in the magnetic field.

Another NMR instrument (Bruker AM-300) was applied to study the effects of a magnetic field on lysozyme crystals in a typical NMR cell [47]. The magnetic field intensity could be as great as 7 T with 99.9% homogeneity. The number of oriented lysozyme crystals decreased as the agarose increased when the crystals were grown in agarose. The number of larger crystals increased when grown in agarose under a homogeneous magnetic field. Subsequently, Surade et al. [48] used an 11.75 T magnet (Bruker NMR 500 MHz) to obtain high-quality crystals of four proteins (lysozyme, thaumatin, ferritin, and INHA-NAD) inside capillary pipettes at a high gel concentration. The transport control induced by gel-growth and kinetic surface control from the magnetic field may contribute to the cooperation observed for this phenomenon.

## 3. Apparatuses for Protein Crystal Growth inside a Magnetic Field

### 3.1. Requirement for Setup Using Protein Crystallization inside the Magnet

A well-designed apparatus is key to growing high-quality crystals. To compare crystals grown in a magnetic field with those grown by conventional techniques, we report in Table 3 the magnetic field intensities with the corresponding resolutions. In consideration of the cost and specificity of the magnetic environment, there have been several requests for the setup to conduct long-duration protein crystallization experiments in a magnetic field.

For the growth of a protein crystal, the equipment is expected to include factors such as convenient crystallization devices, an accurate temperature controlled system, a real-time observation system, and a stable magnetic field. In general, the magnet bore is relatively small to guarantee high magnetic field intensity; therefore, the experimental device must be of limited size (such as a mini crystallization plate). In consideration of the special magnetic properties, the device located within the magnet is expected to consist of a nonmagnetic substance, such as Perspex, ceramic, or bamboo. A real-time temperature monitor is necessary to accurately control the temperature, which is a requirement to grow high-quality crystals and improve the reproducibility of experiments. A real-time observation unit is also necessary to monitor the entire process of protein crystallization. To grow protein crystals, the magnetic field should be sufficiently stable to avoid the influence of other unstable factors (such as mechanical vibration or pressure).

### 3.2. Protein Crystallization Apparatus inside the Magnetic Field

**Figure 5:**
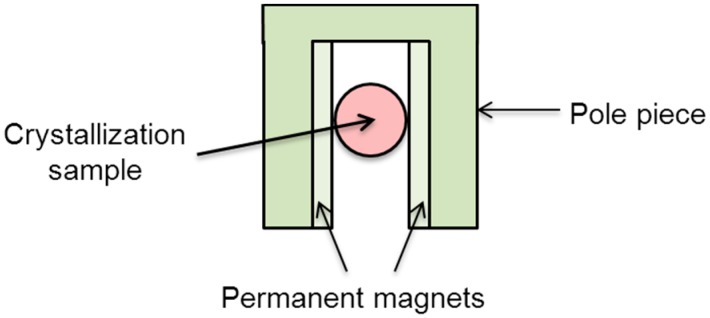
Schematic illustration of the experimental device used in permanent magnets [7].

The development of experimental devices used for protein crystallization inside a magnetic field has been rapid. A simple glass vessel was initially used as the crystallization apparatus. Figure 5 shows a simple experimental device using permanent magnets [7]. The crystallization cell was installed between two small permanent magnets, and the magnets were installed on the microscope by the pole piece to allow the detection of the crystal growth process. This setup allowed observations of the occurrence and growth of crystals by continuous monitoring and recording, but the observation was discontinuous rather than real-time.

**Figure 6:**
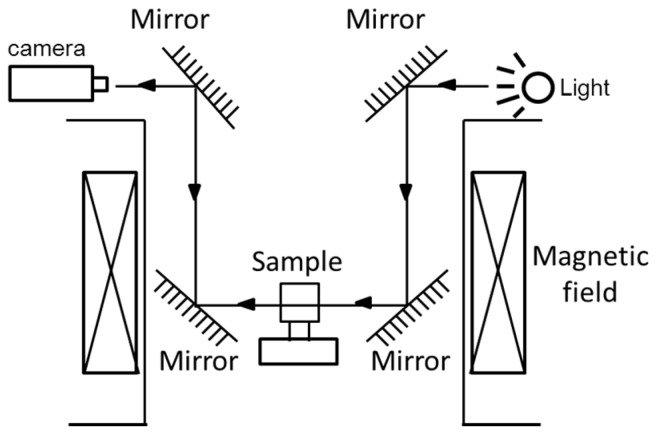
Schematic diagram of the symmetrical device for simultaneously observing protein crystal growth in two locations [51].

This simple glass vessel evolved into an integrative device equipped with a real-time observation apparatus [49], a temperature control system [44], and an auxiliary instrument. Sazaki et al. and Yanagiya et al. [49,50] designed a real-time observation device for use in a strong magnetic field by conveniently adjusting the sample location, as shown in Figure 6. White light was used to illuminate the protein crystallization drops through an optical fiber. The location of the drops could be adjusted by sliding the stages in the *X*, *Y* and *Z* directions. Long-working-distance lenses, a CCD camera, and a videotape recorder were used to capture high-resolution crystal images. The crystallization temperature was controlled by a Peltier element and circulating water. The elements of the experimental device were all made of paramagnetic and diamagnetic materials. The microscope system was installed in the superconducting magnet [22]. A similar in situ observation apparatus was created using two completely identical pieces of equipment positioned symmetrically to record the growth process of protein crystals [51]. The equipment consisted of a light, four mirrors, a zoom lens (Pentax, Tokyo, Japan), and a CCD camera (XC-77, Sony, Tokyo, Japan). The control of crystallization temperatures was recorded at three different positions near the samples by T-type thermocouples. High-quality lysozyme crystals were obtained in a complete levitating state in the device. The advantage of the former apparatus was that the sample position could be adjusted continuously, while the advantage of the latter was the simultaneous observation of a sample in two locations.

**Figure 7:**
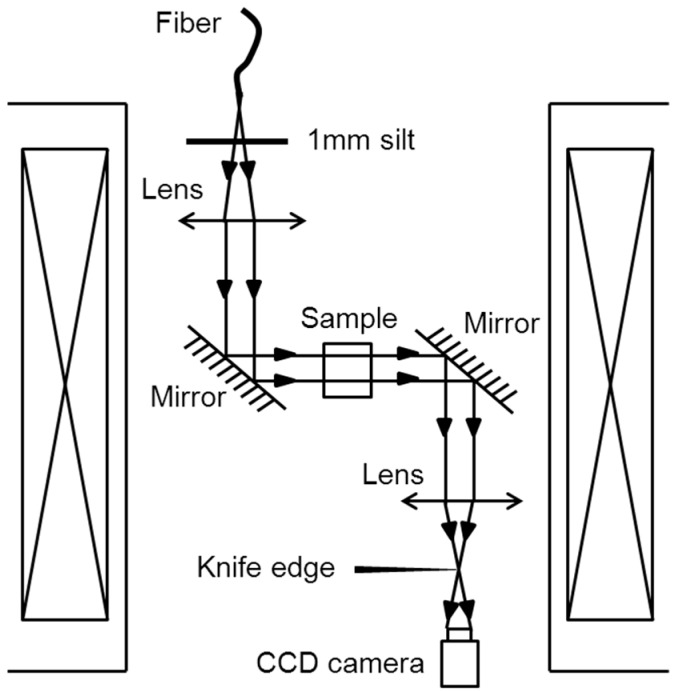
Schematic drawing of the Schlieren microscope in a magnetic field [52].

Poodt et al. [52] designed a long working schlieren microscope in a 20 T resistive magnet (at Radboud University, Nijmegen, The Netherlands), as shown in Figure 7. The coolant was projected on a 1 mm slit, and the slit was placed in the focal plane of an achromatic lens. Using two mirrors, the beams were guided through a glass cuvette with the sample. Finally, the images were recorded by a projective zoom lens and CCD camera system. Using this microscope, the features of protein crystal growth in a gradient magnetic field were studied to compare how the same process occurred in microgravity. The authors showed that gradient magnetic fields can simulate microgravity crystal growth in space.

Another real-time visualization installation for crystal growth in a superconducting magnet was constructed by Okada et al. [53]. The setup consisted of a temperature control system, crystallization cells and an observation system. The crystallization cells were positioned in a plate around a periscope to effectively save space. It is worth noting that the cell plate contained buffer solutions to control the evaporation rates of the crystallization solutions. All of the crystallization cells were composed of optical transparent plastic for optical observation, and they were stacked vertically in the magnetic cavity. Using the observation system (including a periscope, CCD camera and PC), crystals in the cells outside of a magnetic field could be observed. The crystallization temperature was maintained at 4–20 °C by flowing dry, warm air from the bottom. During the experiments, the location of the periscope could be conveniently moved by motors to observe crystal growth.

**Figure 8:**
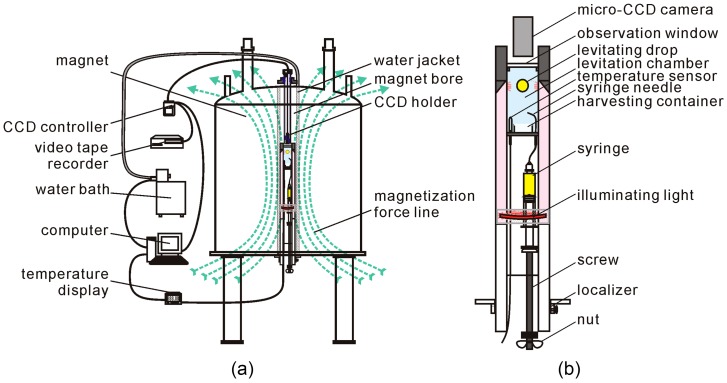
Schematic illustration of a container-less levitation device assembled in a superconducting magnet [44]. (**a**) The containerless levitation setup; (**b**) the levitation unit. Reproduced from reference [44] with permission from AIP Publishing LLC and Copyright Clearance Center.

Lu et al. [44] designed a container-less device for the levitation of crystallization droplets inside of a superconducting magnet (JMTA-16T 50 MF), as shown in Figure 8. The levitation apparatus was composed of a temperature control unit, a levitation unit, and a real-time observation unit [44]. The temperature control unit included a water bath, a temperature sensor, a water jacket, a computer and a temperature control program. A micro-CCD camera was used to observe the levitation process. This easy and simple levitation setup could accurately control the temperature and sample volume. The levitation processes were as follows: first, tightly screwing the screw caused the droplet to grow gradually along with the injection from the syringe; and finally, the droplet would fully levitate in the chamber when the magnetization force was larger than the gravitational force. The crystal growth process of the levitated droplet was observed using this setup, and the lysozyme crystals were obtained in levitation droplets. Shared instruments using the same superconducting magnet (JMTA-16 T 50 MF) were designed by Wang et al. [54]. These instruments included an operating platform, temperature controller, sample holders, and observation system. An outstanding advantage of this apparatus was that it could simulate different gravity levels by orienting the sample at different positions in the magnet. This setup provided a simple, quick and accurate experimental environment in which to conduct research or practical applications, in areas such as life sciences or materials sciences, in a superconducting magnet.

Cooling crystallization, namely the separation of crystals from the crystallization solution caused by lowering the temperature, also plays a vital role in growing protein crystals using a magnetic field. A cooling crystallization instrument was designed by Gao et al. to study the influence of a low magnetic field on the crystal habit, metastable zone, and crystal form [55]. The cooling process was realized by a two-bladed stirrer (speed of 350 rev∙min^−1^). The crystallization temperature was monitored by a programmable thermostat (CKW-2200).

**Figure 9:**
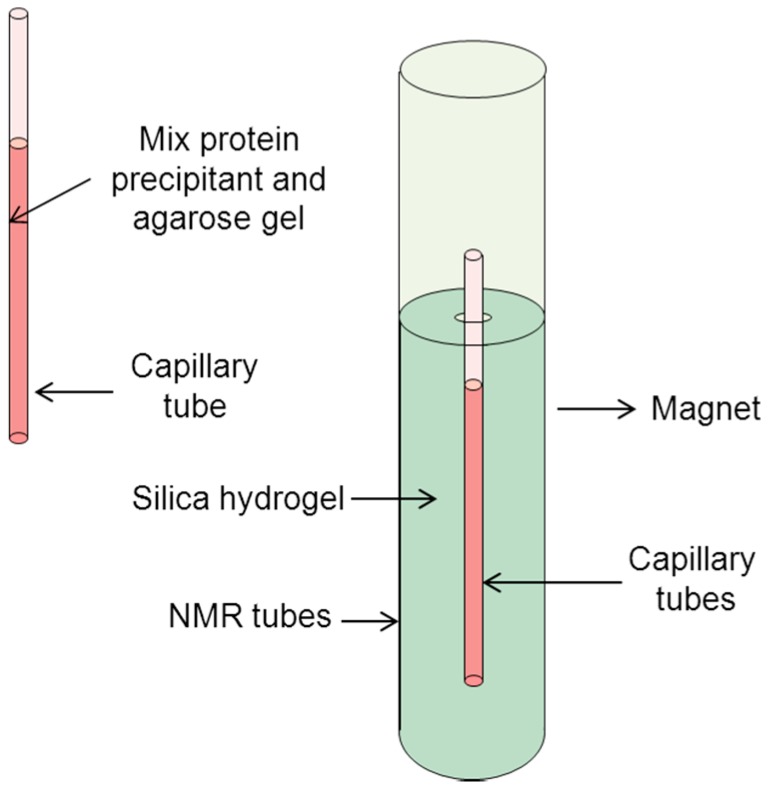
Schematic drawing of the experimental device for protein crystallization in gels. The gels were placed within capillary tubes located inside NMR tubes in magnetic fields [45].

Moreno et al. [45] designed an experimental setup to study protein crystallization in an NMR magnet using the batch method, as shown in Figure 9. In the experiments, protein samples were located in an agarose gel, which was within a capillary surrounded by silica gel to avoid spinning of the capillary. All of the capillary tubes and the silica gel were placed inside the NMR tubes. Using this device, the crystal size, number and alignment of single ferritin, lysozyme, thaumatin and crystals were analyzed.

### 3.3. Other Equipment Used for Protein Crystals under a Magnetic Field

In addition to the classical apparatus used for protein crystallization inside a magnetic field, there have also been excellent designs for detecting parameters such as crystal dissolution, natural convection, and solution viscosity during the growth of protein crystals.

**Figure 10:**
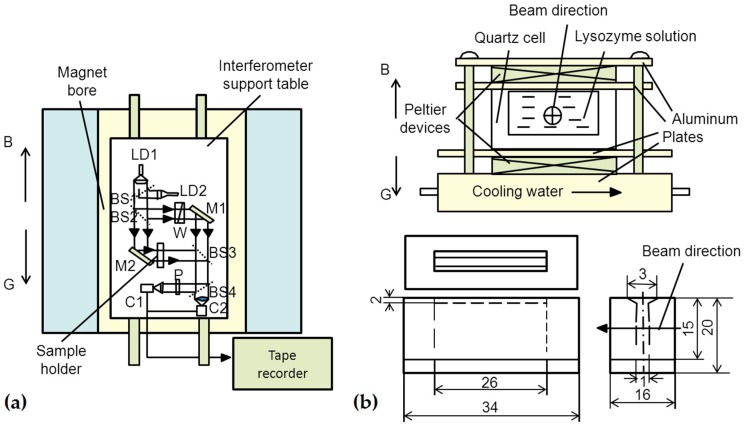
(**a**) Schematic drawing of the Mach–Zehnder interferometer in a superconducting magnet bore; and (**b**) sample holder and enlarged drawing of a sample cell [56]. Reproduced from reference [56] with permission from Elsevier and Copyright Clearance Center.

Initially, Yin et al. [56,57] studied lysozyme crystal growth and dissolution in a uniform magnetic field in situ using a Mach–Zehnder interferometer. The Mach–Zehnder interferometer mainly consisted of two LED lights, four beam splitters, two mirrors and a polarizer, as shown in Figure 10. The two light sources were used for observing the interference image and bright image. The facility was sufficiently stable to resist mechanical vibration, and the interference fringe could be easily adjusted. The authors showed that the crystal growth rate was slower in magnetic fields compared with controls, particularly under heated conditions. The dissolution of lysozyme crystals was suppressed by the magnetic field due to the decreased diffusion coefficient of lysozyme molecules.

**Figure 11:**
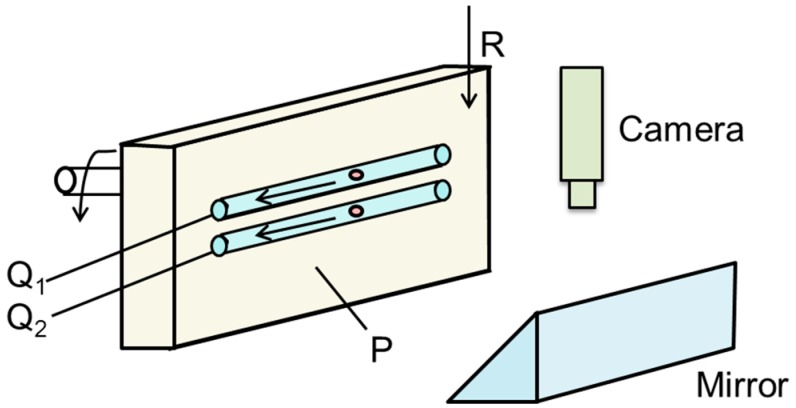
Schematic drawing of the apparatus for measuring viscosity in a superconducting magnet [58].

An apparatus for measuring lysozyme solution viscosity in a magnetic field (10 T) was designed by Zhong et al. [58]. They fixed two plastic tubes on a board and placed one glass ball in each tube, as shown in Figure 11. One tube was filled with a supersaturated protein aqueous solution, and another tube was filled with pure water as a control. The entire setup was placed at the center of the magnet. The authors analyzed the solution’s viscosity by inclining the tube and observing the movement of the glass balls. They showed that the viscosity of a filtered solution was almost independent of the magnetic field strength. However, the viscosity of an unfiltered solution increased when the magnetic field strength was increased, and it returned to the initial value when the magnetic field was turned off. If the magnetic field was maintained for a long time, the solution’s viscosity could not return to the initial value. Zhong et al. [59] investigated correlations between the birefringence of protein solutions, the strength of the magnetic field, and the concentration of a solution under a magnetic field. The experiment was primarily based on two polarizers that were orthogonal to each other. Light intensity was chosen as the target parameter, and magnetic birefringence could be deduced using a formula. The experiment showed that the birefringence of solutions increased under a magnetic field of 10 T, which indicated molecular alignments along the magnetic field direction in protein solutions.

**Figure 12:**
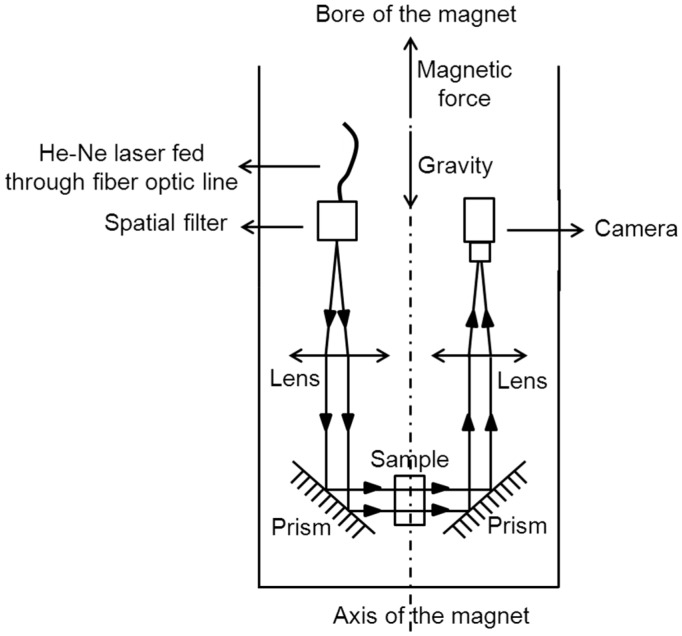
Schematic illustration of the experimental setup for the real-time observation of solution convection [60].

A system for the real-time observation of solution convection was designed by Ramachandran et al. [60] to validate their theoretical prediction. Their instrument included a test cell and an optical system, as shown in Figure 12. In the apparatus, a He-Ne laser passed through a fiber optic line, spatial filter, lens and prism to the test cell, and a similar optical transmission system was fabricated to image the shadowgraphs of the laser to the CCD camera. The test cell was made of machined Plexiglas and three infusion ports. The authors showed that the magnetic field could disturb the locally sedimentation induced by gravity, which was highly consistent with their theoretical prediction.

**Figure 13:**
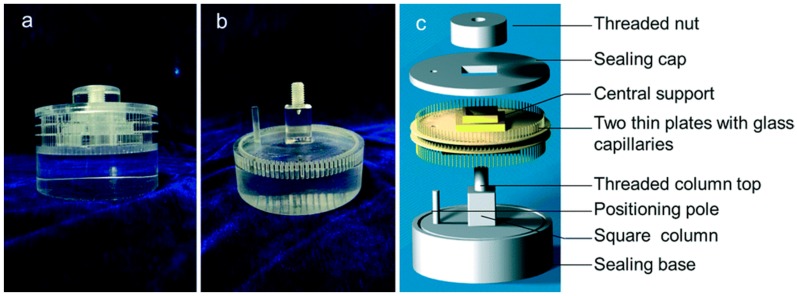
Schematic diagram of the high-throughput crystallization screening setup [26]. (**a**) The high-throughput crystallization setup using in the magnet; (**b**) the reservoir base; (**c**) the structure details of the high-throughput crystallization setup. Reproduced from reference [26] with permission from the PCCP owner societies.

A high-throughput protein crystallization screening instrument under a cryogen-free superconducting magnet was invented [26], as shown in Figure 13. The screening device consisted of 96 capillary tubes of the same length, corresponding to 96 crystallization conditions. The operation process was as follows. First, the capillaries were filled with dry protein. Second, the capillaries were assembled into two thin plates, and the capillaries were filled with screening reagents. Third, the setup was placed in a magnetic field to grow crystals. Using this setup, the in situ diffraction pattern of lysozyme crystals could increase to 2.8 Å in a home facility. The advantages of the device were that it allowed crystallization conditions to be evaluated without removing or cryopreserving crystals and the structure of crystals to be observed without harvesting them.

Nakamura et al. [61] designed an apparatus for in situ and real-time observations of the growth of high-quality protein crystals under quasi-microgravity. The apparatus consisted of an inverted periscope, a temperature control unit, a crystallization plate and other equipment. Time-lapse pictures of protein crystals were captured, and the crystal size, quality and structure were compared. The results showed that the trigonal monomeric Kusabira–Orange crystals were oriented and the *c*-axis of the crystals was parallel to the magnetic field direction. This phenomenon might derive from the diamagnetic anisotropy of protein molecules, especially from the aromatic rings of amino acid residues and planar peptide bonds. In addition, crystals grown in a magnetic field exhibited higher and more homogeneous quality compared with control crystals.

## 4. Future Prospects and Concluding Remarks

Magnetic fields have become a useful tool in protein crystallization. They can unquestionably affect both the crystallization processes and the quality of protein crystals. By taking advantage of the special effects caused by the application of a magnetic field, we hope to be able to adjust the properties of the final crystallization product or investigate the mechanism by which protein crystallization occurs in this unique environment, with the goal of elucidating phenomena that cannot be observed in other environments. Because studies on the effects of magnetic fields on protein crystallization largely rely on the conditions of the hardware, the development of magnet technology is expected to have a strong impact on this research field. Currently, the most widely utilized magnets in research are superconducting magnet systems that can provide a sustainable and stable magnetic field up to 27 T or higher (>30 T) at a reasonable operating cost. However, such magnet systems are usually expensive and may pose problems with convenient access for some small laboratories. Thus, other alternatives are recommended. For instance, a permanent magnet can be considered as one solution to this problem. A permanent magnet can provide a strong magnetic field up to 4 T or higher, depending on the structural design of the assembled permanent magnets. Due to the low cost of the hardware, the almost negligible running cost, and easily implemented protocols, a permanent magnet could potentially serve as a routine condition for protein crystallization under a magnetic field in small laboratories. For studies requiring a higher magnetic field, a superconducting magnet is still the optimal equipment. National magnet laboratories hosting a large number of superconducting, resistive or hybrid magnets are located in several countries, such as the US, Japan, The Netherlands, France, and China. The facilities of these laboratories are usually open to the world and are, in fact, accessible to most interested researchers. With the development of magnet technology, various magnetic field conditions will provide new opportunities for protein crystallization studies. For example, sustainable pulsed magnetic fields are now readily accessible, and due to the effects of pulsed magnetic fields on solutions, it can be anticipated that this special environment may reveal some new phenomena. However, protein crystallization has yet to be studied under such a condition.

Apart from magnet systems, the apparatus utilized in the magnetic field is also important. The most important aspect of protein crystallization is the controllability of the environment. Parameters of the physical environment in a strong magnetic field, such as temperature, pressure, humidity, and acoustic, optical, and electrical parameters, can be incorporated into the package of controllable parameters. In addition to physical parameters, manipulation of the sample (such as rotation, translational movement, or vibration) during the crystallization process in a magnetic field is sometimes necessary. In the era of automated nanoliter technology, it is necessary to consider creating a small, automated-system-applicable vapor diffusion crystallization plate that is useable in a magnetic field to allow sample consumption to be significantly decreased. All of these parameters or manipulations will require special hardware designs to realize their controllability. Furthermore, observation of the crystallization process is also important. The incorporation of a microscope or another probing instrument, such as static/dynamic light scattering, shadowgraph, or interferometer, can be very helpful for the study of the crystallization process.

In this review, we provided an overview of the hardware utilized for studying protein crystallization in a magnetic field. Hardware development is expected to continuously move forward to satisfy new research purposes. With the rapid development of magnet technology, we expect that various magnet systems will be more readily available for protein crystal growers in the near future. Furthermore, beyond protein crystallization, research using magnetic fields in life sciences, materials sciences, physics and chemistry will also require the design and manufacture of hardware similar to that reviewed here. Hence, this review may serve as a useful reference for broader research areas.

## Figures and Tables

**Table 1:** Different magnets used for protein crystallization.

Magnets	Flux Density (T) (Accessible Range)	Features	References
Electromagnets	0–2.4 (0–2.0)	Low cost; continuous function in the range of hours	[6]
Permanent magnets	0.1–1.25 (0.1–4)	Negligible cost; prolonged durability	[7]
Hybrid magnets (resistive magnets)	33–45 (11–45)	Large power consumption; continuous function in the range of hours	[8,9]
Superconducting magnets	4–16.1 (4–27)	Low running cost; sustainable working state for years	[10,11]

**Table 2:** Superconducting magnets used for protein crystallization.

Magnets	Intensity (T)	Manufacturer	Types of Protein	References
Liquid-helium-free superconducting magnet	11	Institute for Materials Research, Tohoku University	Lysozyme, horse spleen ferritin	[22,23,24]
Cryogen-free superconducting magnet	8	Sumitomo Heavy Industry	Lysozyme microcrystals	[25]
Cryogen-free superconducting magnet	12	OXFORD Instruments, UK (Abingdon, UK)	Lysozyme	[26]
Low-temperature superconducting magnet	5	Japan Magnet Technology, Inc. (Kobe, Japan)	l-alanine	[10]
Liquid-helium-free superconducting magnet	10	Japan Magnet Technology, Inc.	Human estrogenic 17 β-hydroxysteroid dehydrogenase, lysozyme snake muscle fructose-1,6-bisphosphatase, bovine adenosine deaminase	[27,28,29,30,31,32,33,34,35]
Low-temperature superconducting magnet	16.12	Japan Superconductor Technology, Inc. (Hyogo, Japan)	Lysozyme, light-harvesting complex 2, protein K, concanavalin, HSP90^N^ thaumatin, catalase, trichosanthin	[11,36,37,38,39]
Laboratory-size superconducting magnet	15	Japan Superconductor Technology, Inc.	Lysozyme, acylphosphatase, nucleoside diphosphate kinase, ST0811, monomeric sarcosine oxidase, flap endonuclease 1	[40,41,42,43]

**Table 3:** A resolution comparison of crystals grown inside and outside of the magnetic field.

Protein	Resolution (Å) (In the Control)	Resolution (Å) (In a Magnetic Field)	Best Resolution (Å) (In the PDB Database)	Magnetic Field Intensity (T)	Types of Diffractometers	References
Lysozyme	1.3	1.13	0.65	10	Rigaku AFC5 four-circle diffractometer	[27]
Bovine adenosine deaminase crystals	2.5	2.0	1.52	10	Rigaku rotating-anode generator	[31]
Lysozyme	1.33	1.13	0.65	10	BL18B at the Photon Factory, Tsukuba, Japan	[35]
Fru-1,6-Pase crystals	3.15	2.9		6 (upper)	Rigaku rotating-anode generator	[29]
		2.9	2.1	10 (middle)		
		6		3.9 (lower)		
Lysozyme	1.20	0.95	0.65	12	Macromolecular Crystallography Beamline (BL17U1) at the SSRF	[36]
Protease K	1.14	0.95	0.98			
Trichosanthin	1.07	1.12	1.6			
Concanavalin A	1.78	1.23	0.94			
Thaumatin	2.70	1.35	0.94			
Catalase	4.64	2.28	0.88			
Heat shock protein90N	2.89	1.61	1.2			
ST0811	1.59	1.10	2.0	8–11	Synchrotron beamlines (SPring-8 or Photon Hyogo, Japan)	[40]
Nucleoside diphosphate kinase	2.61	2.16	1.25			
Flap endonuclease 1	1.90	1.85	1.88			
Pyrococcus horikoshii OT3	1.70	1.50	1.2			
Monomeric sarcosine oxidase	2.70	1.95	1.6			
